# Fabricating high-purity graphite disk electrodes as a cost-effective alternative in fundamental electrochemistry research

**DOI:** 10.1038/s41598-024-54654-0

**Published:** 2024-02-21

**Authors:** Claudia Spallacci, Mikaela Görlin, Amol Kumar, Luca D’Amario, Mun Hon Cheah

**Affiliations:** 1 Molecular Biomimetics, Department of Chemistry - Ångström Laboratory, Uppsala University, Box 523, 75120 Uppsala, Sweden; 2 Structural Chemistry, Department of Chemistry - Ångström Laboratory, Uppsala University, 75121 Uppsala, Sweden; 3 Synthetic Molecular Chemistry, Department of Chemistry - Ångström Laboratory, Uppsala University, 75120 Uppsala, Sweden

**Keywords:** Chemistry, Electrochemistry

## Abstract

Graphite electrodes offer remarkable electrochemical properties, emerging as a viable alternative to glassy carbon (GCE) and other carbon-based electrodes for fundamental electrochemistry research. We report the fabrication and characterization of high-purity graphite disk electrodes (GDEs), made from cost-effective materials and a solvent-free methodology employing readily available laboratory equipment. Analysis of their physical properties via SEM, EDX and XPS reveals no metallic interferences and a notably high porosity, emphasizing their potential. The electrochemical performances of GDEs were found to be comparable to those of GCE. Immobilization of peptides and enzymes, both via covalent coupling and surface adsorption, was used to explore potential applications of GDEs in bioelectrochemistry. Enzyme activity could be addressed both via direct electron transfer and mediated electron transfer mechanism. These results highlight the interesting properties of our GDEs and make them a low-cost alternative to other carbon-based electrodes, with potential for future real-world applications.

## Introduction

Carbon-based electrodes are widely used in various electrochemical applications due to their good electrical conductivity and versatility^1^. In fundamental research, the carbon-based electrodes of choice are glassy carbon electrodes (GCEs) due to their chemical inertness and wide potential window. Besides glassy carbon electrodes, highly ordered pyrolytic graphite (HOPG) is commonly used in electrochemistry research, particularly in protein film electrochemistry, where proteins can be easily immobilized on HOPG electrodes, often without the need for additional surface modification. However, the high cost and difficult fabrication of GCEs or HOPG electrodes precludes their use in real-world applications and these electrodes are often substituted with graphite particles or carbon black-based electrodes. As such, translation from fundamental research to application requires additional optimization due to divergences between the different types of carbon-based electrodes.

As an alternative to GCEs and HOPG electrodes in research laboratories, graphite-based electrodes possess a number of interesting electrochemical properties: excellent conductivity, mechanical stability, chemical inertness, low background current, wide potential window, ease of modification and functionalization^2,3^. These properties make graphite electrodes interesting for a wide range of applications, such as electroanalysis, biosensors, catalysis and energy storage^4–6^.

In literature, there are numerous forms of graphite particle-based electrodes in use. These include screen-printed electrodes (SPEs), carbon paste electrodes (CPEs) and graphite pencil electrodes. SPEs are single-use and formulations of the carbon-based ink for their fabrication are often confidential information; as such, the electrochemical performances of SPEs may vary significantly between different commercial sources. Similarly, the electrochemical performances of graphite pencil electrodes are highly dependent on the formulation of graphite pencil rods, which are not specified by almost all commercial sources. On the other hand, preparation protocols for CPEs in the laboratory are well established and the electrochemical performances of these electrodes only depend on the formulation and purity of the chemicals used in its preparation. However, due to the use of organic liquid binders in CPE, they are only suitable for aqueous environments.

In this paper, we report a simple, solvent-free, procedure for fabricating graphite disk electrodes (GDEs) for use in research laboratories. The only tools required are a moderate temperature oven and a KBr pellet press, which are commonly found in chemistry laboratories. We proceed by (1) characterizing the electrode surface, focusing on its physical properties and morphology (SEM, EDX, XPS); (2) we then test its electrochemical performances, by comparing it to commercial glassy carbon electrodes; (3) we conclude by showing some examples of potential applications in bioelectrochemistry, such as electrode functionalization for peptide coupling and enzyme immobilization.

## Experimental section

### Reagents and chemicals

All chemicals were of analytical grade. Polyethylene (PE), 4-aminophenylacetic acid, sodium nitrite (NaNO_2_), hydrochloric acid, tetrabutylammonium tetrafluoroborate (TBABF_4_), tetrabutylammonium hexafluorophosphate (TBAPF_6_), acetonitrile (ACN), potassium ferrocyanide, potassium ferricyanide, hexaamineruthenium(III) chloride, ferrocene, ferrocenemethanol, p-benzoquinone, potassium chloride (KCl), N-hydroxysuccinimide (NHS), N(3-dimethylaminopropyl)-N′-ethylcarbodiimide hydrochloride (EDC), L-cysteine, Glucose oxidase (GOD) from *Aspergillus niger* and Catalase from bovine liver were purchased from Sigma Aldrich Sweden AB. Natural graphite particles (spherical graphite, 99.95% C, 10 μm particle size) were purchased from ProGraphite GmbH, Germany. 4-Carboxybenzenediazonium tetrafluoroborate was synthesized following a literature procedure^7^. Bilirubin oxidase (BOD) was provided by Nicolas Mano^8^.

### Solutions and buffers

Phosphate buffer solutions (NaPi, KPi) were prepared by mixing either sodium or potassium monobasic dihydrogen phosphate and dibasic monohydrogen phosphate, and then adjusting the pH. MES buffer was prepared by dissolving 2-(N-morpholino)ethanesulfonic acid to the desired concentration and adjusting the pH. Electrochemical assays were performed in 1 mM potassium ferricyanide in KPi buffer with the addition of 1 M KCl, 1 mM hexaamineruthenium(III) chloride in KPi buffer, 1 mM ferrocene in ACN with 0.1 M TBAPF_6_ previously bubbled with Ar gas, and 1 mM p-benzoquinone in ACN with 0.1 M TBAPF_6_ previously bubbled with Ar gas. 2 mM 4-Carboxybenzenediazonium tetrafluoroborate in ACN with 0.1 M TBAPF_6_ as supporting electrolyte was used for surface functionalization of the electrodes. Activation and coupling solutions were freshly prepared before use, by mixing 20 mM EDC and 4 mM NHS in MES buffer. 5 mM L-cysteine solution was prepared in MES buffer. BOD stock solution was prepared by dissolving 5 µL of a 0.38 mM concentrated BOD solution in 400 µL of NaPi buffer. GOD stock solution was prepared by dissolving 5 mg of GOD in 1 mL of NaPi buffer. Catalase stock solution was prepared by dissolving 1.3 mg of lyophilized catalase in 1 mL NaPi buffer.

### Instrumentation

A conventional kitchen spice grinder was used for grinding PE flakes before mixing with graphite powder. 13 mm diameter graphite disk electrodes were obtained using a Specac^®^ Atlas 15T manual hydraulic press and a Specac^®^ macro–micro KBr pellet die. A Metrohm Autolab potentiostat was used for electrochemical measurements. A Biologic SP-300 potentiostat was used for EIS measurements. SEM images were acquired using a ZEISS LEO 1550 scanning electron microscope, which was equipped with an InLens detector and operated at an acceleration voltage of 3 kV. Energy dispersive X-ray spectroscopy (EDX) was collected at 15 kV using the AZtec software from Oxford Instruments. XPS data were obtained using a PHI Quantera II Scanning XPS Microprobe located at Myfab Uppsala. To evaluate the specific surface area of the electrodes, N_2_ adsorption isotherms were measured at 77 K using a Micromeritics ASAP 2060 micropore physisorption analyzer. Prior to measurement, electrodes were activated at 160 °C overnight, using a Micromeritic FlowPrep 60 sample preparation unit; Brunauer–Emmett–Teller (BET) method was used to calculate the specific surface area. A custom-designed Clark-type sensing electrode was used for O_2_ detection.

### Electrode fabrication

GDEs with different PE %w were compared; this study focuses on GDEs with 15% and 25% PE, since 5% and 35% PE GDEs were also tested and did not show satisfactory results (Fig. S1, S2). The fabrication protocol consists of the following steps: (1) PE flakes were ground through a conventional spice grinder into smaller particles; (2) A weighted amount of ground PE was mixed with natural graphite powder and transferred to a glass vial; (3) The vial was placed on a vortex mixer for 30 s, four times; (4) The vial was left on a rotate/tumble machine overnight (15 h) to obtain a homogenous powder mixture; (5) The powder mixture was incubated at 200 °C for 2 h in an oven and cooled to room temperature; (6) 200 mg of product were put into a 13 mm pellet press and vacuum was applied for 2 min; (7) Disks were obtained by pressing at 10 tons for 3 min under vacuum. The final disks have a diameter of 13 mm and are approximately 1 mm in thickness. The material cost for a 13 mm diameter 25% PE GDE is estimated to be €0.0324 per electrode (€0.0244/cm^2^), which is very cost-effective when compared to, for example, carbon fiber paper^9^ with a cost of €0.1030/cm^2^.

### Electrochemical measurements

All experiments were performed with a Metrohm Autolab potentiostat at room temperature, using a custom-designed low-volume electrochemical cell (Fig. S3). The diameter of the exposed working electrode area is 5 mm for all measurements. For measurements in aqueous solution, a graphite pencil rod was used as counter electrode. An Ag/AgCl (in 3 M KCl) electrode was used as reference and regularly checked using K_4_[Fe(CN)_6_] as standard (E°_Ag/AgCl_ = + 0.210 V vs. SHE). EIS measurementd were performed using a Biologic SP-300 potentiostat, in NaPi buffer, with the same electrochemical cell setup, in the frequency range 0.1–200 kHz (logarithmic spacing) and an amplitude of 10 mV. The results were modeled with conventional Cole elements. The measurements in organic solvent (acetonitrile) were performed with 0.1 M TBAPF_6_ as supporting electrolyte. A graphite pencil rod was used as counter electrode. The reference electrode was made by an Ag wire coated with AgCl and sealed in a glass tube fritted with Vycor porous glass tip and filled with the same electrolyte solution. All potentials are reported versus Ag/AgCl. For measurements in acetonitrile, potentials are internally referenced to the ferrocene couple (Fc^+^/Fc), then converted to Ag/AgCl using the factor E°_Fc_+_/Fc_ = + 0.501 V versus Ag/AgCl. For all measurements, the glassy carbon working electrodes were polished with 0.05 μm alumina particles and sonicated for 3 min in distilled water immediately before use. The graphite disk electrodes were polished on P2000 grit sandpaper and sonicated for 3 min in distilled water immediately before use.

### Electrode functionalization

Electrodes were functionalized with surface 4-phenylacetic groups via electrochemical reduction of the corresponding benzyl diazonium salt, with a protocol adapted from literature^10^: cyclic voltammetry was recorded in 2 mM 4-carboxybenzenediazonium tetrafluoroborate in acetonitrile (0.1 M TBAPF_6_ supporting electrolyte) and cycled from + 0.6 to − 0.7 V ten times. The electrodes were rinsed with pure ACN, dried and finally rinsed with MES buffer.

### Peptide and enzyme immobilization

Activation of superficial carboxyl groups was performed according to the literature^8^, by soaking the electrodes in 1 mL of 20 mM EDC and 4 mM NHS in MES buffer for 1 h at room temperature under gentle shaking. The electrodes were rinsed with MES buffer. Coupling of L-cysteine was performed by soaking the activated electrodes in 2 mL of a 5 mM L-cysteine solution in MES buffer overnight at 4 °C. The electrodes were rinsed and stored in MES buffer at 4 °C. Enzyme immobilization was performed via covalent coupling or via physical adsorption. Coupling of BOD was performed by soaking the previously functionalized and activated electrodes in 100 µL BOD stock solution and 400 µL NaPi buffer at 4 °C overnight. Adsorption of BOD was performed by soaking the bare electrodes in 100 µL BOD stock solution and 400 µL NaPi buffer at 4 °C overnight. Adsorption was also tested on bare GDEs and GCEs by dropcasting 30 µL of a 0.08 mM solution of BOD on the bare electrodes and drying; measurements were performed after thorough rinsing with NaPi buffer. Coupling of GOD was performed by soaking the electrodes in 20 µL GOD stock solution and 300 µL NaPi buffer at 4 °C overnight. Adsorption of GOD was performed in the same conditions as coupling, but using functionalized electrodes and skipping the activation and coupling steps. The electrodes were rinsed and stored in NaPi buffer at 4 °C when not in use. O_2_ detection experiments with a Clark-type electrode were performed in the same electrochemical cell, containing 2 mL NaPi stirred solution. An applied potential of − 700 mV was applied to the Clark electrode. On the bottom of the cell, a GDE or GCE was placed after GOD immobilization. 5 µL of catalase solution (1.3 mg/mL) were added to the system, immediately followed by 11 µL of 2.22 mM glucose solution. Oxygen consumption was recorded over time.

## Results and discussion

### Physical properties

**Figure 1:**
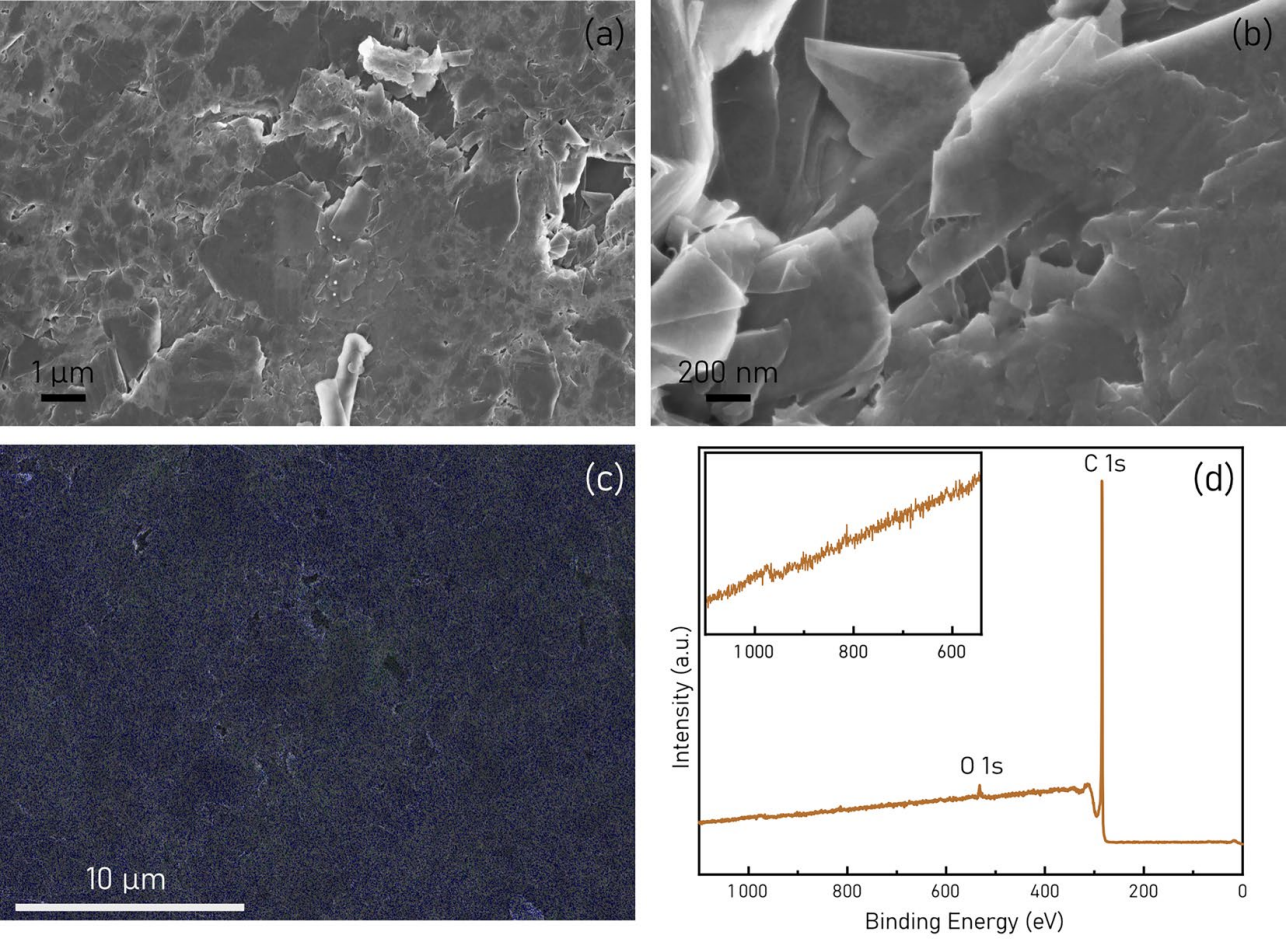
Analysis of the physical properties of the GDEs. (**a**–**b**) SEM images of the GDE. (**c**) EDX elements mapping on the 20% PTFE GDE surface; blue = C, green = F. (**d**) XPS survey spectra of 25% PE GDE after polishing.

Graphite disk electrodes (GDEs) were fabricated according to the procedure reported above. Polyethylene (PE) was added to highly pure graphite powder to increase the robustness of the electrode disk. Thorough polishing of pure graphite GDEs would also be more challenging. Effects of PE addition on the electrochemical performance are discussed later (Section “Electrochemical impedance spectroscopy” in “Electrochemical performances”). Newly fabricated GDEs are robust and easy to handle. GDEs with 15% PE are mechanically softer compared to GDEs with 25% PE. In both cases, a smooth and homogeneous surface is obtained after polishing. To assess the efficiency of the mixing procedure and the homogeneity of the graphite-polymer mix, special GDEs were prepared following the same procedure, but replacing PE with 20% w/w polytetrafluoroethylene (PTFE). SEM analysis (Fig. 1a,b) was performed to characterize the microscopic structure of the electrode surface, while EDX was able to map the presence of fluorine to assess the polymer coverage after the mixing, melting and pressing procedure. The electrodes display a highly pure, macroporous structure. In Fig. 1c, F is represented with a green color and it is evenly spread on the surface, confirming a high degree of homogeneity of the graphite-polymer mixture.

X-ray photoelectron spectroscopy (XPS) was used to study the surface composition and the potential presence of metals or O-containing functional groups. XPS data confirm the high purity of the graphite surface, since no additional metal ions could be detected (Fig. 1d, zoomed panel). The bare electrode, after polishing, is composed prevalently of C (98.08%), with only a small fraction of O (1.92%). The C 1 s spectra can be deconvoluted into 3 peaks, namely C=C, C–C and C–O, situated at 284.4, 284.7 and 285.5 eV respectively. A small contribution from the π —> π* transition between graphite sheets is visible around 291 eV. The O 1*s* spectra can be fitted with a single peak from C–O at 532.5 eV (Fig. S4).

BET gas adsorption measurements were conducted on 25% PE GDEs, in order to estimate the porosity of the material and quantify the surface area available to gas permeation (Table S1). The average BET surface area calculated is 5875 cm^2^ for the full disk with a geometric area of 3.062 cm^2^; this means that the area available for gas adsorption is ~ 1900 times higher than the geometric area available. It is important to note that this area will not be entirely available to the electrolyte solution during the electrochemical measurement, due to the different permeability of gas and liquids.

### Electrochemical performances

The electrochemical performances of the GDEs were tested and compared with those of a commercial glassy carbon electrode plate (GCE). The same electrochemical cell configuration was used, so that the geometric area of GCE and GDE exposed to the electrolyte solution is 0.196 cm^2^ (d = 5 mm) in both cases.

#### Electrochemical impedance spectroscopy

To test the behavior of the electrodes upon PE addition, Electrochemical Impedance Spectroscopy (EIS) was performed on the GDEs and GCE (Fig. S5, Table S2). The measurements were carried out in NaPi buffer, at − 0.35 V, 0.15 V and 0.65 V. At all potential values, the 25%PE GDE and GCE show a capacitive behavior (i.e. a constant phase parameter of ca. 0.9). In contrast, the spectrum of the 15% PE GDE exhibits a mixed behavior, in which a diffusion component arises. As a reference, the measurement was performed on a pure graphite GDE: in this case, the EI spectrum is dominated by diffusion limited processes, with a classic Warburg line in the Nyquist plot (constant phase parameter ca. 0.5). The capacitance of the pure graphite GDE is ca. three orders in magnitude higher than that of the other electrodes. The large capacitance of the pure graphite GDE can be ascribed to the high porosity of the material. However, this morphology also induces a strong dynamic diffusion limitation, as suggested by the Nyquist plot. This behavior is not observed in the 15% PE and 25% PE GDE, in which the graphite-PE composite limits the access to the inner pores of the electrode, resulting in a capacitive behavior comparable to that of glassy carbon (GCE).

#### Electrochemical potential window

The electrochemical stability of an electrode in a given electrolyte is of practical significance for potential applications. To estimate the potential window for operativity of the GDEs compared to commercial GCEs, cyclic voltammetry was recorded in aqueous solution with phosphate buffer and MES buffer and in organic solvent using acetonitrile (Fig. S6). In the aqueous solvents tested, the potential window of the GDEs is generally comparable to that of a commercial GCE. More specifically, in phosphate buffer the electrochemical window is in the range of − 1.5 to 1.5 V, while in MES buffer the 25% PE GDE seems to have a slightly wider electrochemical window than the GCE (− 0.6 V to 0.9 V) and the 15% PE GDE (− 0.4 V to 0.8 V). We note that for aqueous solutions, all three electrodes require electrochemical pretreatment by running cyclic voltammetry at reducing potentials for at least five cycles and Ar sparging to obtain background traces without any appreciable O_2_ reduction response (Fig. S7). In acetonitrile, the cathodic limit of the GDEs is reached already at − 1.5 V, while GCE is stable until − 2.0 V; no significant oxidative current is observed until 1.5 V. However, the 15% PE GDE exhibits a significant increase in capacitive current, whereas the 25% PE GDE remains comparable to the GCE, in accordance with the EIS analysis. Generally, the wettability of GCEs is greatly improved in aqueous solutions, whereas the graphite surface of GDEs is highly hydrophobic, resulting in enhanced wettability in organic solvents.

#### Electron transfer properties

**Figure 2:**
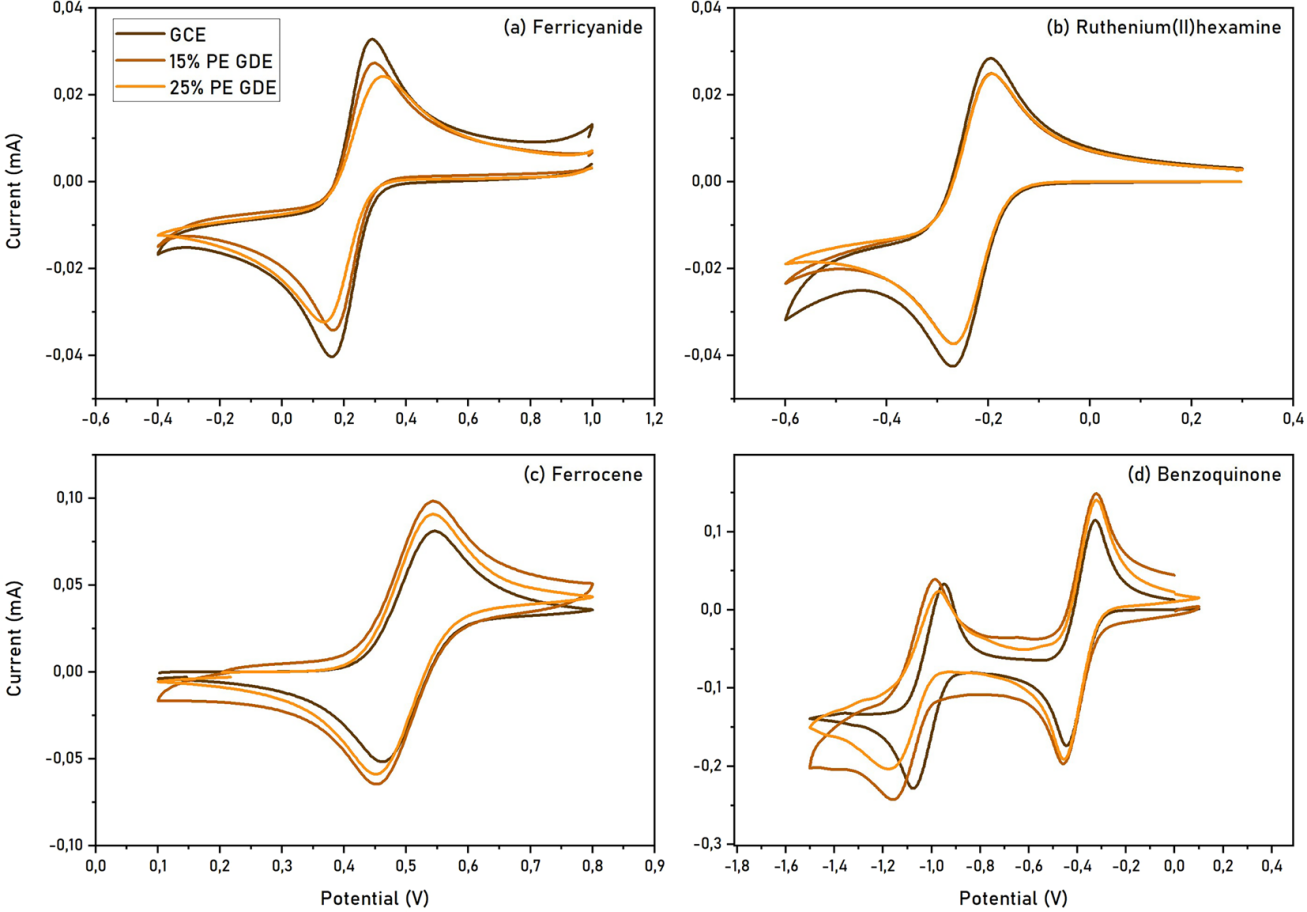
Cyclic voltammetry measurements of different redox probes for GCE, 15% PE GDE and 25% PE GDE. (**a**) 1 mM potassium ferricyanide in phosphate buffer 0.1 M, pH = 7, 1 M KCl; (**b**) 1 mM ruthenium(II) hexamine in phosphate buffer 0.1 M, pH = 7; (**c**) 1 mM ferrocene in 0.1M NBu_4_PF_6_ in acetonitrile; (**d**) 1 mM benzoquinone in 0.1 M NBu_4_PF_6_ in acetonitrile.

The electron transfer properties of the polished electrodes were analyzed by cyclic voltammetry, monitoring the oxidation and reduction current peaks of well-known redox probes at different scan rates. The selected redox probes are potassium ferricyanide, ruthenium(II) hexamine, ferrocene and benzoquinone (Fig. 2).

Based on the voltammograms, the electron transfer efficiency of GDEs is generally comparable to that of GCEs. In aqueous solution, the GCE seems to display higher peak currents, but the peak-to-peak separation is more similar to the ideal case for the 15% PE GDE in phosphate buffer; in MES buffer, the two GDEs show similar performances. In organic solvent, the GCE displays lower currents. This behavior reflects what was observed above regarding the higher hydrophobicity of the GDEs compared to commercial GCEs, resulting in enhanced wettability in organic solvent and thus higher currents. Testing benzoquinone electrochemistry in acetonitrile (Figs. 2d, S8), it is interesting to note that the second reduction step displays a higher capacitive current and a slight loss in reversibility for the GDEs compared to GCE; anomalies have already been observed in literature and rationalized in different ways, such as the effect of the supporting electrolyte, the presence of water impurities, and possible side reactions following the first electron transfer^11–13^. In the case of the GDEs, this deviation could be attributed to the higher porosity of graphite electrodes compared to GCE and to the likely presence of OH groups on the surface, which can act as proton donors in quinone radical reduction^14^.

The Randles–Sevcik equation (Eq. 1) describes the behavior of the peak current for reversible electron transfer processes (at 298 K)^15^:

1 $$I_{p} = \, \left( {2.69 \, \times \, 10^{5} } \right)n^{3/2} ACD^{1/2} \nu^{1/2}$$

where *C* is the concentration of the redox probe (1 mM), *A* is the electroactive surface area (EASA, cm^2^), *D* is the diffusion coefficient of the redox probes (cm^2^ s^−1^) and *ν* the scan rate (V s^−1^). Knowing the diffusion coefficients of the redox probes (for anodic current in aqueous solution, respectively 6.4 × 10^−6^ cm^2^/s for ferricyanide and 7.86 × 10^−6^ cm^2^/s for ruthenium(II) hexamine)^16^, it is possible to obtain values of EASA (Table 1, Figs. S9–S10).

**Table 1:** Values of faradaic EASA calculated using anodic currents and double-layer capacitance for the different electrodes.

Electrode	Faradaic EASA (cm^2^)		C_DL_ (µF)
	Ferricyanide	Ruthenium(II) hexamine	
GCE	0.165	0.111	58.76
15% PE GDE	0.122	0.117	24.40
25% PE GDE	0.095	0.114	9.69

It is interesting to notice that in presence of ferricyanide, a decreasing value of EASA is observed following the trend GCE > 15% PE GDE > 25% PE GDE (respectively 0.165, 0.122 and 0.095 cm^2^). Comparing the faradaic EASA to the geometric surface area (0.196 cm^2^), this corresponds to a decrease in the surface available for electron transfer from 85 to 63% to 49% respectively for the three electrodes. According to these results, the EASA of the 15% PE GDE appears to be 1.3 times higher than the 25% PE GDE EASA. For ruthenium(II)hexamine, comparable values of EASA, respectively 0.111, 0.117 and 0.114 cm^2^ for GCE, 15% PE GDE and 25% PE GDE were calculated, indicating that the presence of different %PE in the melt does not significantly affect the faradaic response. These values correspond on average to 58% of the geometric surface area available. These results are highly dependent on the efficiency of the electron transfer from the electrode surface and the specific redox species present in solution; for this reason, the different characteristics of the GCE and GDEs surface (*e.g.* the presence of different surface groups depending on the applied potential) and the ionic charge of the selected redox species need to be considered.

#### Double-layer capacitance

The values of electrochemically active surface area obtained with the method above are largely affected by the efficiency of the electron transfer at the electrode surface to a target analyte. Measurements of EASA using the Gouy-Chapman theory of double-layer capacitance are also typically employed. This method has been shown to be strictly dependent on the applied potential, electrolyte concentration and electrode roughness^17,18^. However, deriving EASA values based on this methods requires pre-knowledge of the specific capacitance of the electrode material, which is not available for the GDEs. Nonetheless, we obtained double-layer capacitance values following the method reported by Jaramillo^19,20^, in a solution containing only supporting electrolyte. The potential range is chosen as a 100 mV interval in which no faradaic current is present. Cyclic voltammetry measurements were conducted by sweeping the potential across the region from negative to positive potential and back at different scan rates: 0.05, 0.01, 0.025, 0.05, 0.075, 0.1 and 0.2 V s^−1^. The working electrode was held at each potential vertex for 10 s before beginning the next sweep. All measured current in this non-Faradaic potential region is assumed to be due to double-layer charging. The electrochemical double-layer capacitance, C_DL_ (F), was estimated at 0.15 V by plotting the double-layer charging current i_c_ as a function of the scan rate, ν (V s^−1^), as given by (Eq. 2):

2 $$i_{c} = \nu C_{DL}$$

The plots yield a straight line, with slope corresponding to the double-layer capacitance (Fig. S11, Table 1). Comparing the two GDEs, it is interesting to note that an increase in %PE, from 15 to 25%, corresponds to a decrease in double-layer capacitance of a factor of 2.5 (Table 1). However, for the same GDEs, this change in %PE leads to a decrease in faradaic EASA by a 1.3 factor (see above, in presence of ferricyanide). Overall, this translates into a better sensitivity of the 25% PE GDEs, due to the higher ratio of faradaic versus non-faradaic (capacitive) current. These results are in line with the EIS analysis mentioned above: the higher double-layer capacitance of the 15% PE GDE is accounted for by the higher capacitive resistance and mixed capacitive-diffusion behavior observed (Table S2).

### Potential applications

To test the potential applications of our GDEs in bioelectrochemistry, surface functionalization was carried out to address covalent attachment of peptides and proteins. The GDEs were functionalized with superficial 4-phenylacetic groups via electrochemical reduction of the corresponding benzyl diazonium salt. A surface coverage of 2.6 × 10^–9^ mol cm^−2^ was estimated by integration of the reduction wave upon release of N_2_ gas. XPS analysis confirmed the presence of carboxyl moieties at 289 eV (Fig. S12a). Carbodiimide chemistry was chosen for covalent coupling of peptides, and L-cysteine was used to probe the success of the reaction. Coupled L-cysteine on GDEs showed an intense peak in the S 2p XPS region, at 167 eV; control experiments with dropcasted L-cysteine on the electrode surface resulted in only a slightly visible S 2p peak (Fig. S12b).

To further analyze the behavior and electron transfer properties of the GDEs towards immobilization of enzymes, Bilirubin oxidase (BOD) and Glucose oxidase (GOD) were chosen. These two enzymes can be addressed via two different mechanisms: direct electron transfer (DET) from the electrode surface to the catalytic center of the protein in the case of BOD; mediated electron transfer (MET) in the case of GOD, using ferrocenemethanol as a redox mediator^21^. Immobilization of the enzymes was achieved via covalent coupling in the same conditions described above and compared to surface adsorption.

**Figure 3:**
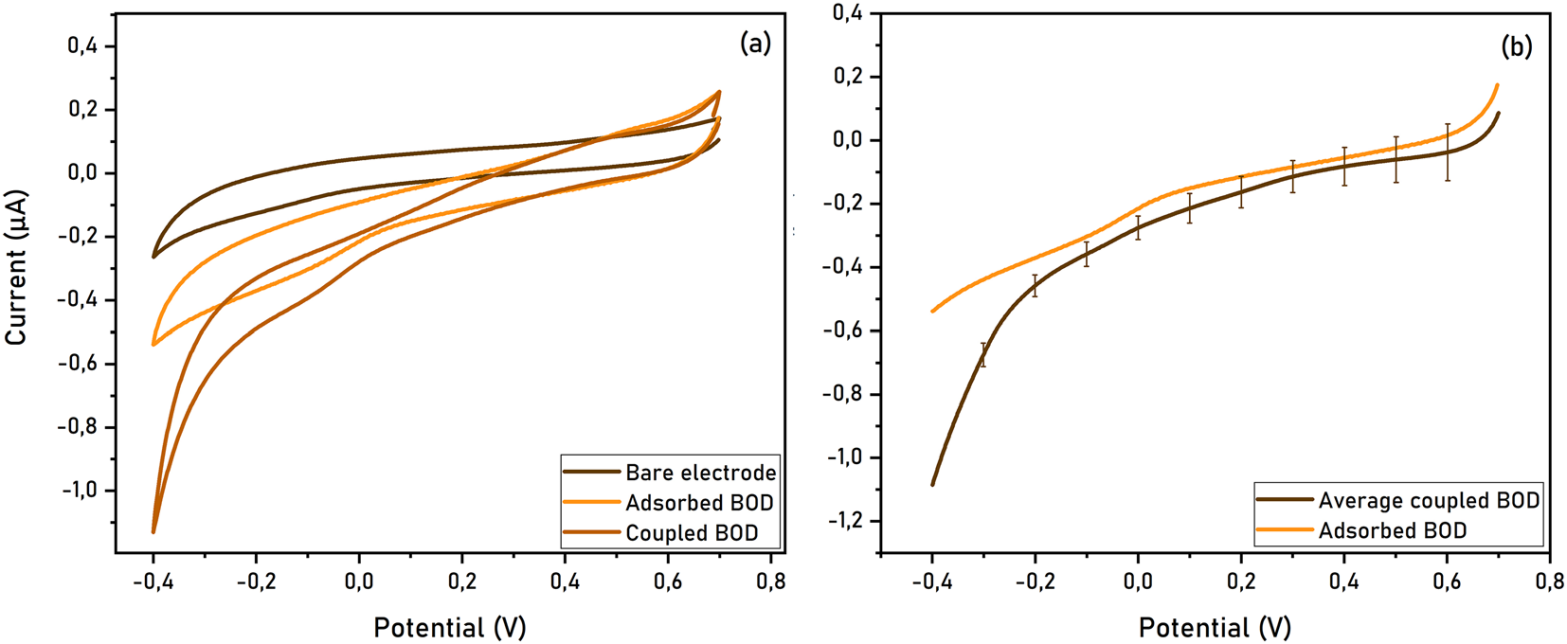
Electrochemical analysis of BOD immobilized on GDEs. (**a**) Cyclic voltammetry measurements of covalently coupled and adsorbed BOD on GDE (phosphate buffer pH 6.8, scan rate 10 mV s^−1^). (**b**) Average of multiple reduction scans for coupled BOD compared to adsorbed BOD on GDEs.

Bilirubin oxidase is a well-studied enzyme and it has been previously addressed electrochemically due to its ability to receive electrons via a DET mechanism. The T1 Cu center, which binds and oxidizes the substrate in the natural enzyme, is located at 7–8 Å from the protein surface; it is believed that this site is the target of efficient interfacial electron transfer from electrodes biased at a sufficient reducing potential. Electrons are then transferred to the T2/T3 Cu site, where O_2_ is reduced^22–24^. Both covalent coupling and physical absorption of BOD on GDEs show a catalytic wave with the same onset potential at 0.47 V versus Ag/AgCl, confirming the suitability of these electrodes to electrochemically address immobilized enzymes (Fig. 3). Furthermore, higher reducing currents are obtained in the case of covalently linked enzymes, indicating that this procedure leads to higher enzyme loading on the electrode surface compared to absorption in the same conditions.

Dropcasting of BOD on GDE or GCE shows a clear catalytic response for both electrodes. Even though a different shape of the catalytic wave is observed, the same onset potential and comparable cathodic currents at higher reductive potentials indicate the suitability of both electrodes for this type of measurement (Fig. S13).

**Figure 4:**
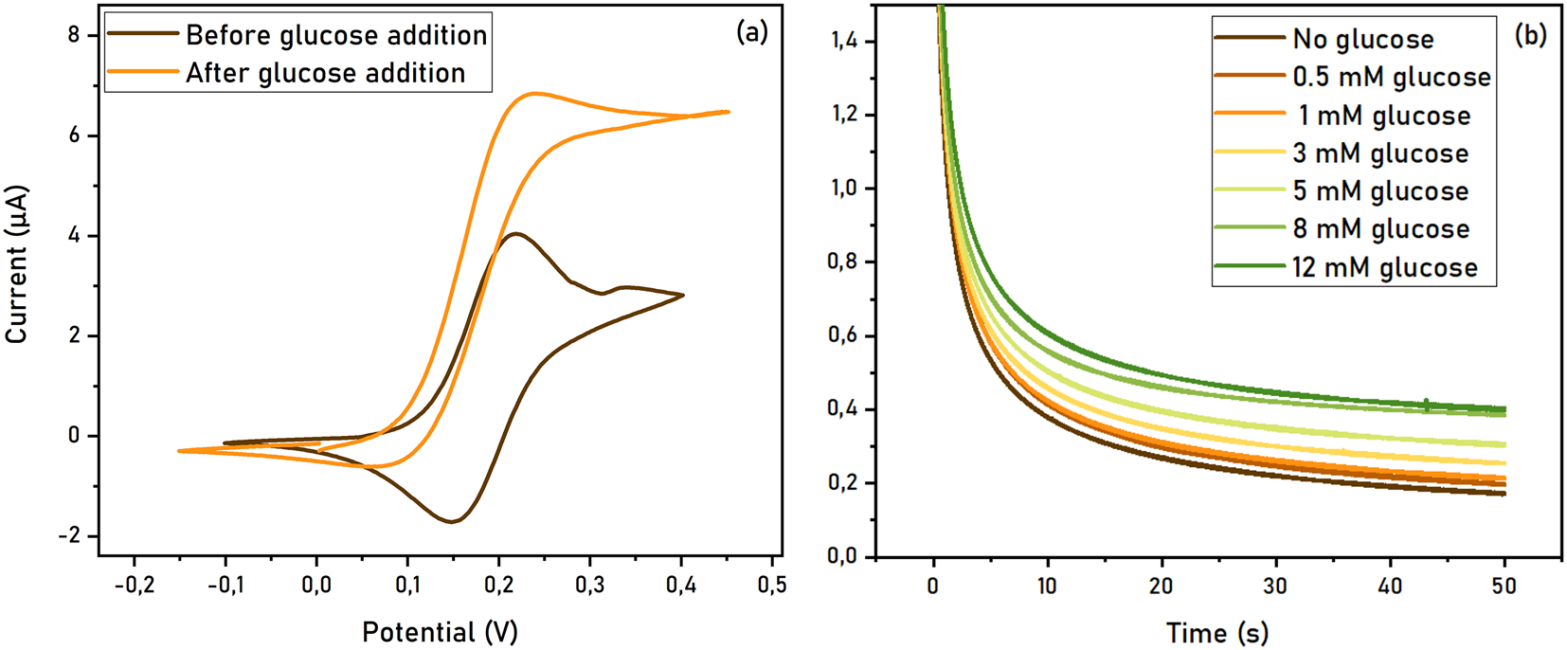
Electrochemical analysis of GOD immobilized on GDEs. (**a**) Cyclic voltammetry measurements of covalently coupled GOD on GDE (phosphate buffer pH 6.8, scan rate 1 mV/s). (**b**) Chronoamperometry performed at 0.25 V with addition of increasing amounts of glucose.

Glucose oxidase (GOD) immobilization was also tested. GOD has been extensively studied in the last decade and has currently many applications in food and beverage processing, pharmaceutical, chemical, medical diagnostics, biotechnology, clinical chemistry, environmental protection, energy and textile industries^25^. GOD is a flavoprotein that oxidizes β-D-glucose to D-glucono-δ-lactone, using oxygen as an electron acceptor and releasing H_2_O_2_. Although some studies claiming DET mechanism have been published^26^, the enzyme has been mainly addressed and utilized relying on mediated electron transfer mechanism^21,27^. In this study, ferrocenemethanol (Fc) was selected as a redox mediator for its solubility in aqueous solution and because the potential of the ferrocenemethanol/ferroceniummethanol (Fc/Fc^+^) couple is high enough to ensure the oxidation of the enzyme (Scheme S1). GOD was immobilized on the electrode surface via covalent coupling, as described above. In absence of substrate, the cyclic voltammogram shows the characteristic shape of the reversible Fc/Fc^+^ couple. Upon glucose addition, a sigmoidal shape of the voltammogram is obtained, typical of the presence of a catalytic process: the height of the anodic peak undergoes a large increase while the cathodic peak decreases (Fig. 4a). This effect is larger with decreasing scan rate and it is in line with previously reported studies^28,29^. Chronoamperometric traces with increasing amounts of substrate performed at a fixed potential of 0.25 V show increasing oxidative current generated from the enzyme (Fig. 4b).

Immobilization via physical adsorption was also tested, but no change in the shape of the voltammogram is observed in this case (Fig. S14), indicating that covalent grafting of this enzyme highly enhances its catalytic properties. The same experiments were performed on GCE, which shows comparable results (Fig. S15). This suggests that both electrodes are suitable to electrochemically address MET of GOD. However, higher catalytic currents reached via covalent coupling on GDE seem to indicate a higher enzyme loading in this case. To further investigate this hypothesis and to test direct activity of the immobilized enzyme, a Clark electrode setup was used to detect oxygen consumption during reaction with glucose. In this measurement, no redox mediator is added, thus a change in oxygen concentration in solution is directly attributable to the enzymatic process. In absence of any bias potential, the addition of glucose to the solution leads to a decrease in oxygen concentration, which is highly enhanced in case of GDE (Fig. S16). This confirms the presence of active enzyme on the electrode surface and of a higher loading on GDE compared to GCE, as discussed above.

## Conclusion

In this work, we have fabricated and characterized high-purity graphite disk electrodes (GDEs) for research laboratory use. The electrodes are made from cost-effective materials and readily available laboratory equipment. Polyethylene (PE) was added to graphite powder, to enhance surface area reproducibility and diminish capacitive currents, ensuring the reliability of the electrodes. The fabrication process is expeditious and straightforward.

We have analyzed the physical properties of the GDEs via SEM, EDX and XPS. The results demonstrate the exceptional purity of the graphite-PE composite. Importantly, no metallic interferences were detected, distinguishing the GDEs' composition as superior to other commercially available carbon-based electrodes, including standard pencil graphite. As such, these electrodes are well-suited for research in catalysis, where the presence of adventitious metal contamination often leads to false positive results. Furthermore, Brunauer–Emmett–Teller (BET) analysis revealed a notably high porosity in the PE-graphite mixture, highlighting its potential.

The electrochemical performances of the GDEs were compared to those of commercial GCEs. The GDEs revealed a potential window of operativity suitable for a wide range of applications, both in aqueous and organic solvents. Their background capacitance was found to be either comparable or lower than that of GCEs. Cyclic voltammetry experiments employing well-established redox probes confirmed the efficiency of electron transfer at the electrode surface, with rates comparable to GCEs. Interestingly, it was noted that a higher %PE in the graphite-PE composite suppresses capacitance to a higher factor compared to faradaic efficiencies, which remain approximately equivalent: this translates into a better sensitivity of the GDEs.

To test for practical applications of GDEs, immobilization of peptides and enzymes was investigated, both via covalent coupling and surface adsorption. Successful electrode functionalization and covalent coupling were confirmed by XPS data. BOD and GOD were chosen as model enzymes to assess the suitability of GDE to address enzyme activity via DET and MET mechanisms, respectively. In both cases, covalent coupling revealed increased enzyme loading compared to adsorption, leading to enhanced catalytic currents. These results support the potential of our electrodes for use in bioelectrochemistry research, such as protein film electrochemistry or bioelectrocatalysis.

In conclusion, we have successfully developed cost-effective, robust and disposable graphite disk electrodes using a simple, solvent-free fabrication procedure, specifically tailored for research laboratory applications. The straightforward fabrication process and the interesting properties of our GDEs also make them suitable for further refinement and customization for potential real-world applications.

## Supplementary Information

Supplementary Information.

## Data Availability

All data generated or analyzed during this study are either included in this published article and its supplementary information file or are available from the corresponding authors on reasonable request.
