# Correction: Fabricating high-purity graphite disk electrodes as a cost-effective alternative in fundamental electrochemistry research

**DOI:** 10.1038/s41598-026-36170-5

**Published:** 2026-01-15

**Authors:** Claudia Spallacci, Mikaela Görlin, Amol Kumar, Luca D’Amario, Mun Hon Cheah

**Affiliations:** 1 Molecular Biomimetics, Department of Chemistry ‑ Ångström Laboratory, Uppsala University, Box 523, 75120 Uppsala, Sweden; 2 Structural Chemistry, Department of Chemistry ‑ Ångström Laboratory, Uppsala University, 75121 Uppsala, Sweden; 3 Synthetic Molecular Chemistry, Department of Chemistry ‑ Ångström Laboratory, Uppsala University, 75120 Uppsala, Sweden

Correction to: *Scientific Reports* 10.1038/s41598-024-54654-0, published online 21 February 2024.

The original version of this Article contained errors in the Results and discussion section, under the subheading ‘Electron transfer properties’.

“Knowing the diffusion coefficients of the redox probes (for anodic current in aqueous solution, respectively 6.4 × 10^6^ cm^2^/s for ferricyanide and 7.86 × 10^6^ cm^2^/s for ruthenium(II) hexamine)^16^, it is possible to obtain values of EASA (Table 1, Figs. S9–S10).”

now reads:

“Knowing the diffusion coefficients of the redox probes (for anodic current in aqueous solution, respectively 6.4 x 10^−6^ cm^2^/s for ferricyanide and 7.86 x 10^−6^ cm^2^/s for ruthenium(II) hexamine)^16^, it is possible to obtain values of EASA (Table 1, Figs. S9–S10).”

The original Article has been corrected.

