# Supplementary material for: Fabricating high-purity graphite disk electrodes as a cost-effective alternative in fundamental electrochemistry research
Source: Sci Rep. 2024 Feb 21;14:4258. doi: 10.1038/s41598-024-54654-0 (PMC10881485; doi:10.1038/s41598-024-54654-0)
Supplement: Supplementary file 1 — Supplementary Information. [file 41598_2024_54654_MOESM1_ESM.pdf]

Supporting Information

Fabricating high-purity graphite disk electrodes as a cost-effective alternative in fundamental electrochemistry research

Claudia Spallacci*[a], Mikaela Görlin[b], Amol Kumar[c], Luca D'Amario[a], Mun Hon Cheah*[a]

[a] Molecular Biomimetics, Department of Chemistry - Ångström Laboratory, Uppsala University, SE-75120 Uppsala, Sweden

[b] Structural Chemistry, Department of Chemistry - Ångström Laboratory, Uppsala University, SE-75121 Uppsala, Sweden

[c] Synthetic Molecular Chemistry, Department of Chemistry - Ångström Laboratory, Uppsala University, SE-75120 Uppsala, Sweden

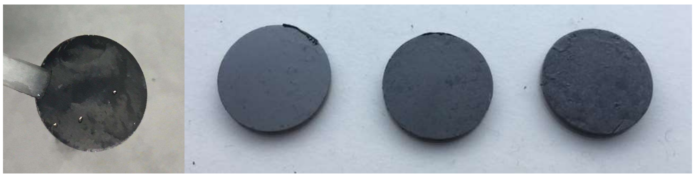

Figure S1. Newly fabricated GDEs of different PE percentages, respectively 5%, 15%, 25% and 35% from left to right.

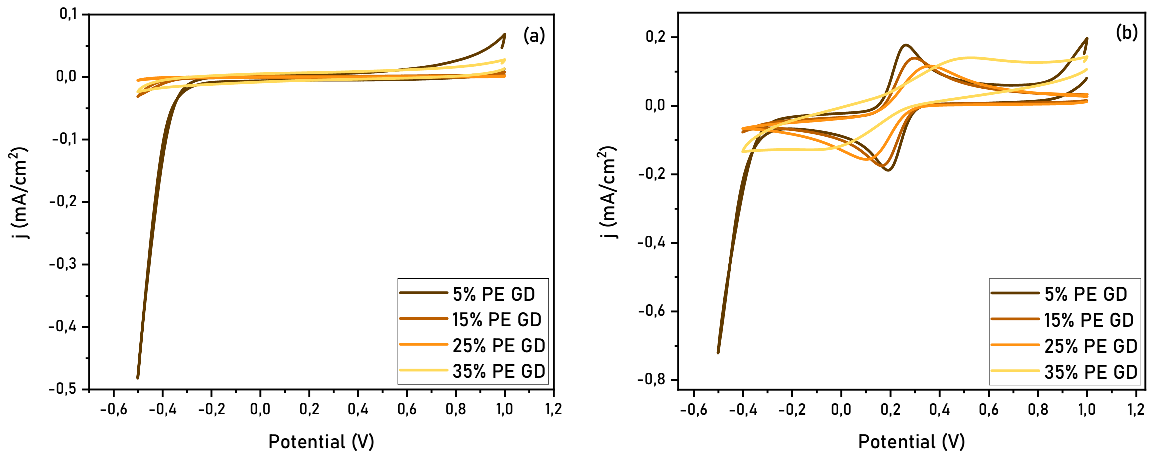

Figure S2. (a) Potential window measurements of 5% PE GDE, 15% PE GDE, 25% PE GDE and 35% PE GDE in phosphate buffer 0.1 M and 1 M KCl, pH = 7. (b) 1 mM potassium ferricyanide in phosphate buffer 0.1 M, pH = 7, 1 M KCl.

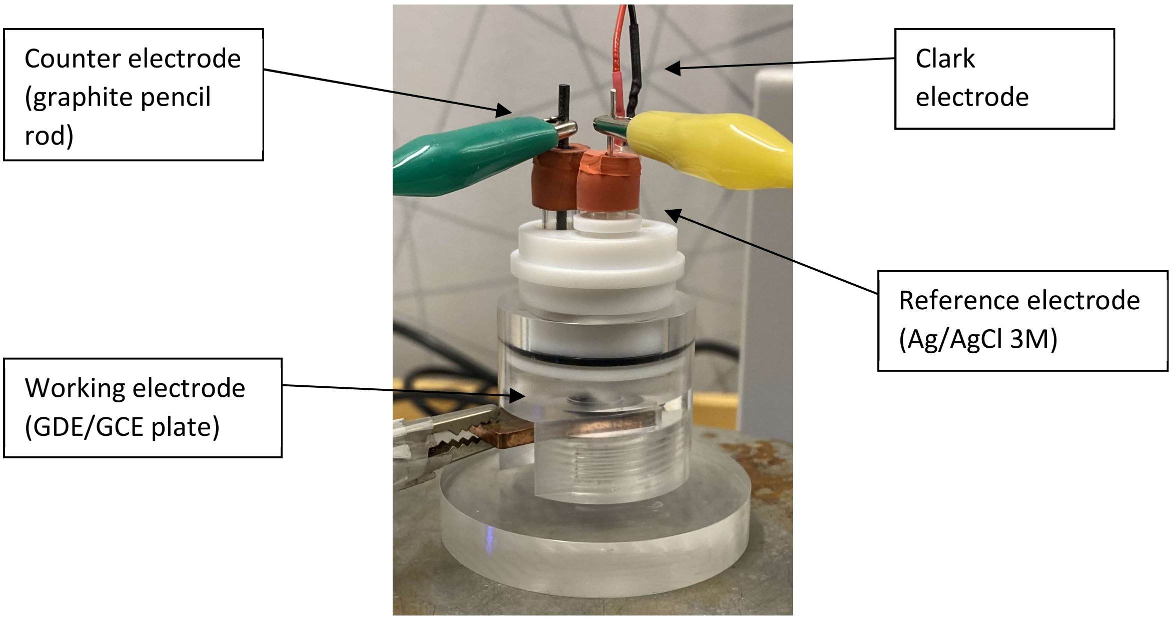

Figure S3. Custom-designed electrochemical cell under operating conditions.

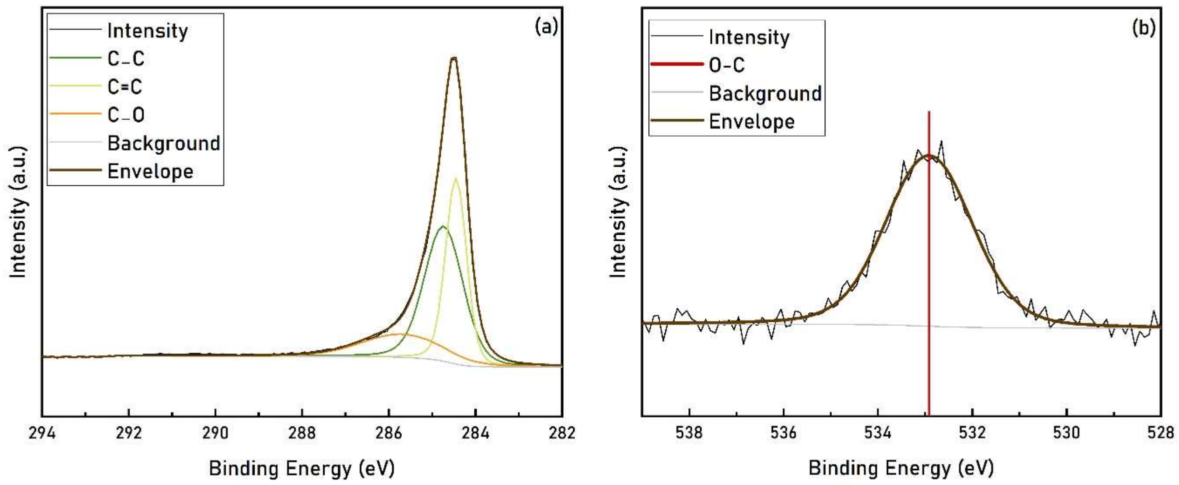

Figure S4. XPS measurements of 25% PE GDE after polishing. High-resolution spectra of the (a) C 1s and (b) O 1s regions.

**Measurement**	**BET Surface Area (cm^2 g^{-1})**	**Sample weight (g)**	**Normalized BET Surface Area (cm^2)**
**1**	79832	0.0861	6874
**2**	56467	0.0864	4879
**Average**			**5856**

Table S1. BET data for 25%PE GDE.

EIS analysis

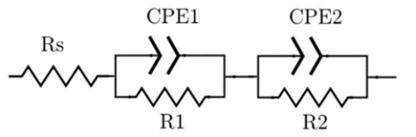

All samples have been fitted with the equivalent circuit above, except for the 15% PE GDE, for which a third Cole element (CPE3 + R3) was required for proper fitting. The resistance Rs originates from the overall cell resistance and is not relevant to the current analysis. The Cole elements consist of a constant phase element, CPE, and a resistance R in parallel. The two fitting parameters for the CPE are the capacitance factor (CPE-T) and the phase factor (CPE-P, ranging from 0 to 1). The results for all electrodes are summarized in Table S2, divided based on circuit element.

Every Cole element represents a charge transfer/accumulation step. The first element accounts for charge accumulation in the double-layer, and, for GCE and 25% PE, it can be fitted with a phase factor of ca. 0.9. In contrast, in the case of the pure graphite GDE, the phase factor becomes close to 0.5, and the Nyquist plot appears as a straight line instead of a semicircle (i.e. a Warburg impedance behavior). In absence of PE, in fact, the electrolyte can penetrate the pores of the material, in which the diffusion of charges is presumably slow. Hence, diffusion processes dominate the impedance signal. The 15% PE GDE exhibits a mixed behavior and would require a more elaborated fit, which goes beyond the scope of our current analysis.

Moreover, the values of R for the first Cole element are all quite large, as expected when no electron transfer occurs (no faradaic processes involved).

In our material, it is reasonable to attribute the second element to the charge hopping between the graphite particles or to the charge transfer between the electrode contacts. This element will contribute to the impedance at high frequency, with negligible values of CPE and R.

Examples of fitting for the 25% PE GDE and the pure graphite GDE, recorded at 150mV, are reported in Figure S5.

		**Rs**	
**Sample**	Potential (mV)	**R**	**%Err**
**Pure graphite GDE**	150	-4723	1160.9
	650	-951.2	432.98
	-350	-354.5	491.06
**15% PE GDE**	150	-524.3	2.3807
	650	-528.8	3.4368
	-350	-532.6	7.8689
**25% PE GDE**	150	-525.8	3.4749
	650	-529.6	3.5434
	-350	-527.4	4.7455
**GCE**	150	-557	3.9876
	650	-546.8	5.8144
	-350	-559.6	4.7841

		CPE1				R1	
Sample	Potential (mV)	T	$T_{\%Err}$	P	$P_{\%Err}$	R	$R_{\%Err}$
Pure graphite GDE	150	0.0038372	1.3349	0.52414	1.1359	51076E+5	35518E+3
	650	0.0038479	0.78929	0.53129	0.7996	1E+20	100
	-350	0.004672	0.40997	0.51497	0.43045	1E+20	100
15% PE GDE	150	6.98E-06	0.79826	0.87394	0.34743	1E+20	100
	650	6.71E-06	0.91211	0.87966	0.48019	1E+20	100
	-350	2.16E-05	3.8696	0.76903	2.3773	60478	8.4103
25% PE GDE	150	1.12E-06	0.77522	0.92468	0.16404	9694100	14399
	650	1.10E-06	0.80144	0.93827	0.1677	5040500	7724
	-350	1.69E-06	1.3055	0.89991	0.27548	478000	2.2946
GCE	150	4.56E-06	1.0821	0.90139	0.26461	1887600	15425
	650	3.30E-06	1.5997	0.91431	0.37355	1395100	12315
	-350	5.29E-06	1.5135	0.90466	0.35743	169890	2.7285

		CPE2				R2	
Sample	Potential (mV)	T	$T_{\%Err}$	P	$P_{\%Err}$	R	$R_{\%Err}$
Pure graphite GDE	150	5.96E-11	2228.7	0.77305	5362	4944	1109
	650	1.05E-09	632.56	0.78203	7.5418	1180	349.04
	-350	4.37E-10	556.01	0.91502	6.3057	524	332.23
15% PE GDE	150	1.94E-09	9.3822	1.05	0.92966	680.4	2.0243
	650	2.06E-09	13261	1044	1.3278	687.7	2902
	-350	2.08E-09	30.38	1042	3.0498	691.2	6.6607
25% PE GDE	150	3.02E-09	11111	1009	1.1951	697.9	2.7288
	650	3.02E-09	11163	1009	1.2046	702	2.7788
	-350	2.83E-09	15198	1015	1.6226	698.3	3.7295
GCE	150	2.3869E-09	12089	1028	1.2864	722.3	3151
	650	2.1703E-09	18.15	1038	1.8962	709	4.6017
	-350	2.4737E-09	14297	1024	1533	726.4	3773

		CPE3				R3	
Sample	Potential (mV)	T	T%Err	P	P%Err	R	R%Err
15% PE GDE	150	1.13E-05	10599	0.76598	1.9635	921.4	5.8226
	650	1.33E-05	19033	0.77811	3.4527	661.8	9.9583
	-350	1.53E-05	51103	0.77966	9.8012	443.1	27249

Table S2. Fitting results for EIS analysis.

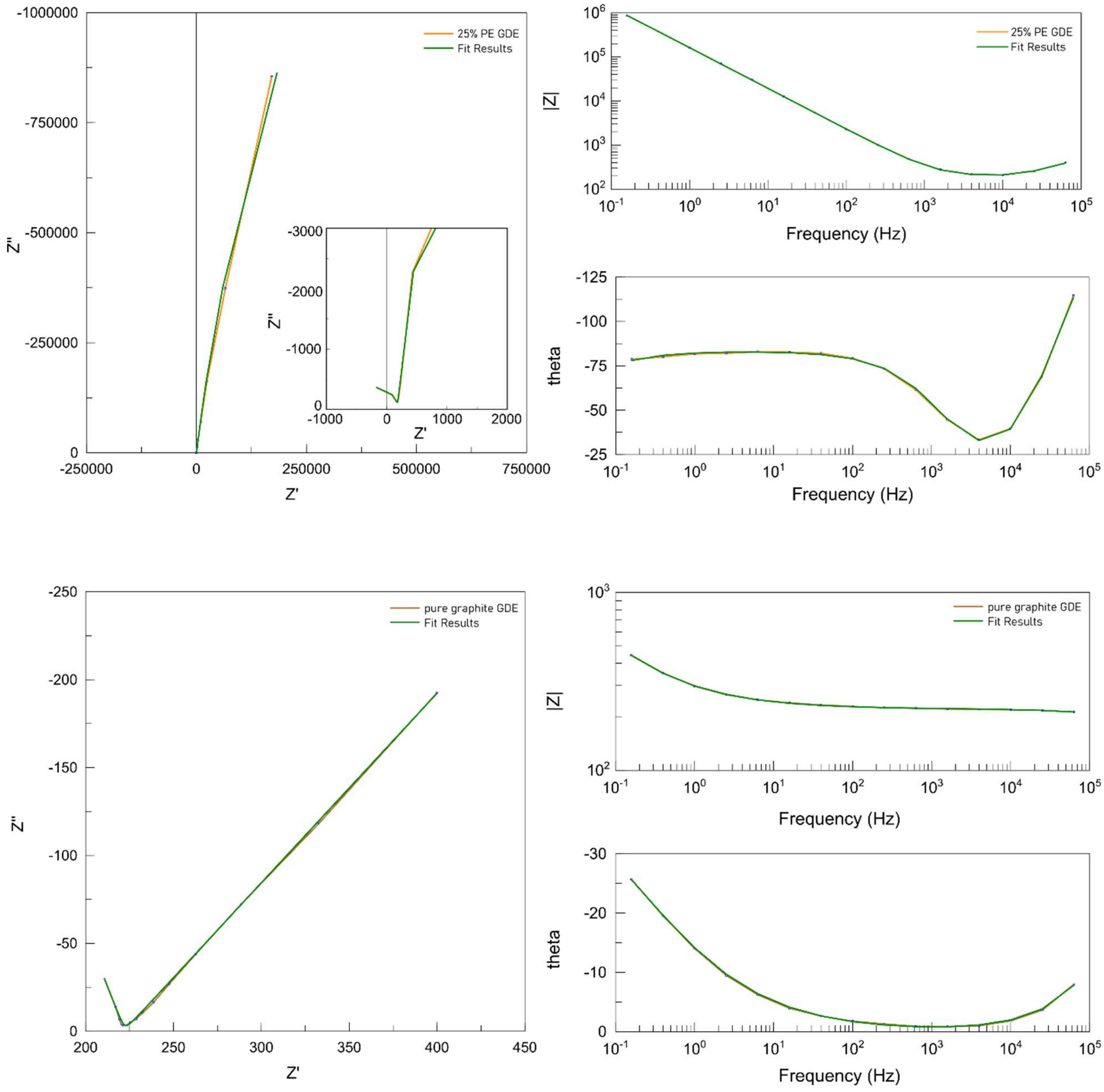

Figure S5. Nyquist plots (left) and Bode phase plots (right) of EIS on 25% PE GDE (top) and pure graphite GDE (bottom).

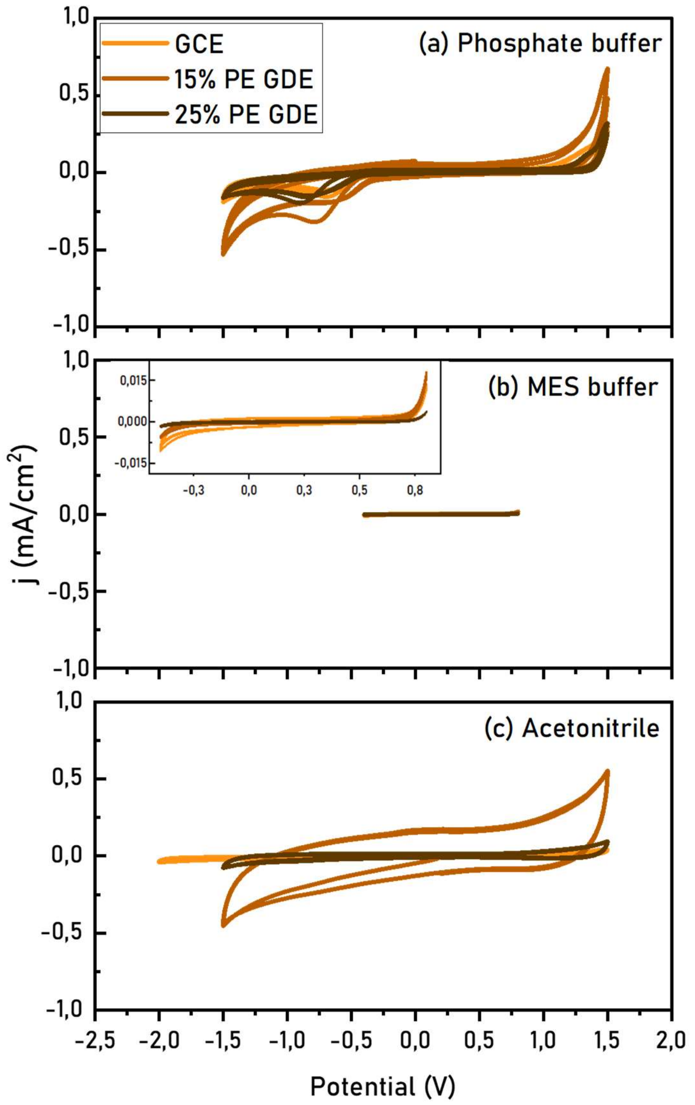

Figure S6. Potential window measurements of GCE. 15% PE GDE and 25% PE GDE for operativity in different electrolyte conditions: (a) Phosphate buffer 0.1 M, pH = 7; (b) MES buffer 0.1 M, pH = 6.5; (c) 0.1 M $TBAPF_6$ in acetonitrile.

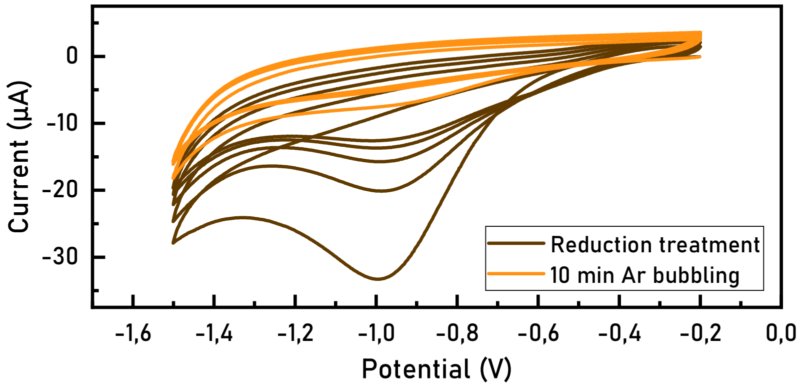

Figure S7. Background traces in phosphate buffer upon reduction and after Ar bubbling, performed right after reduction.

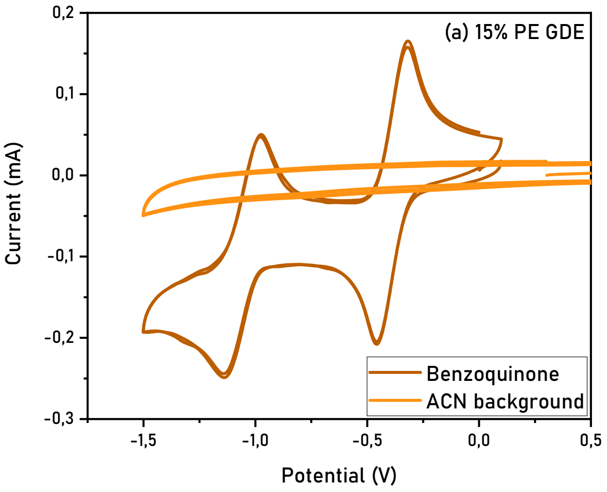

Figure S8. Cyclic voltammetry measurements of 1 mM benzoquinone in 0.1M NBu_4PF_6 in acetonitrile for (a) 15% PE GDE and (b) 25% PE GDE.

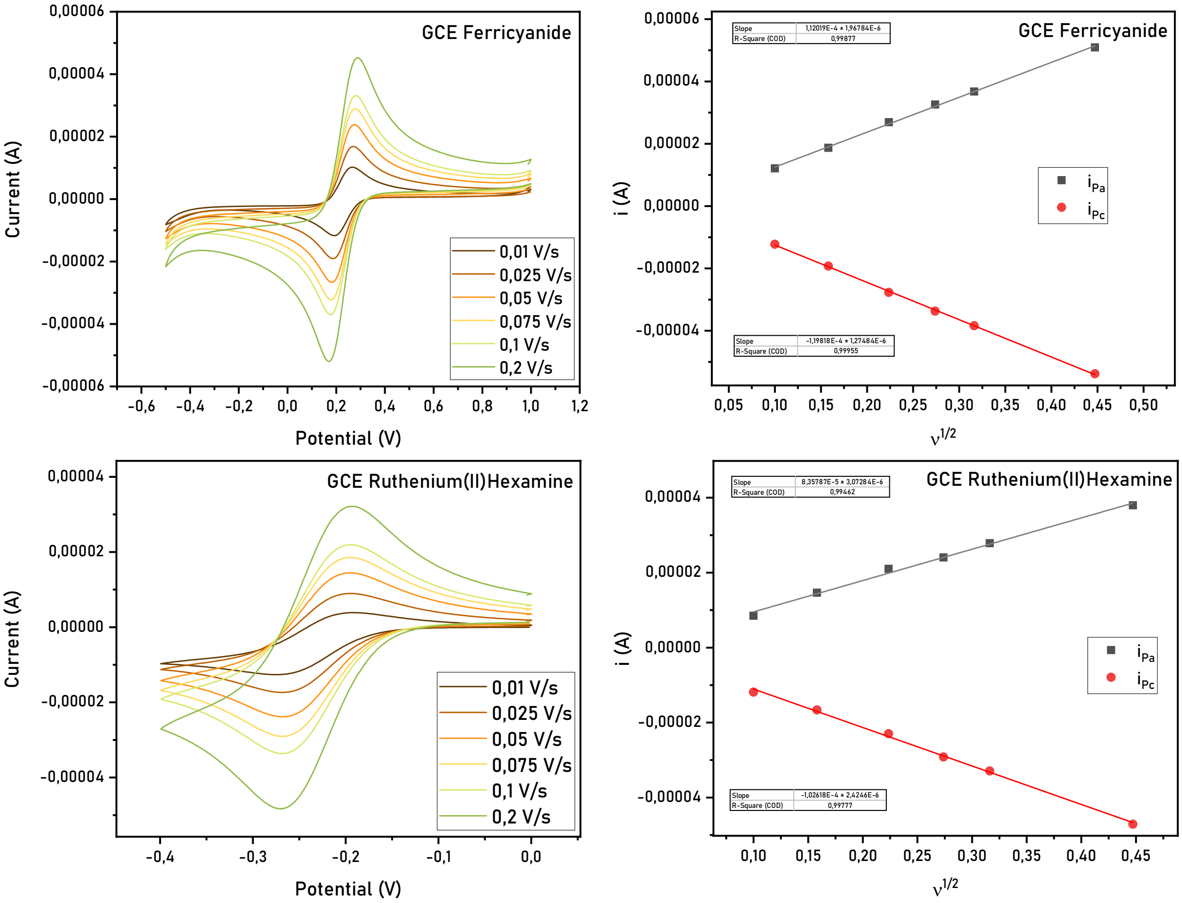

Figure S9. Example of faradaic EASA experiment for GCE.

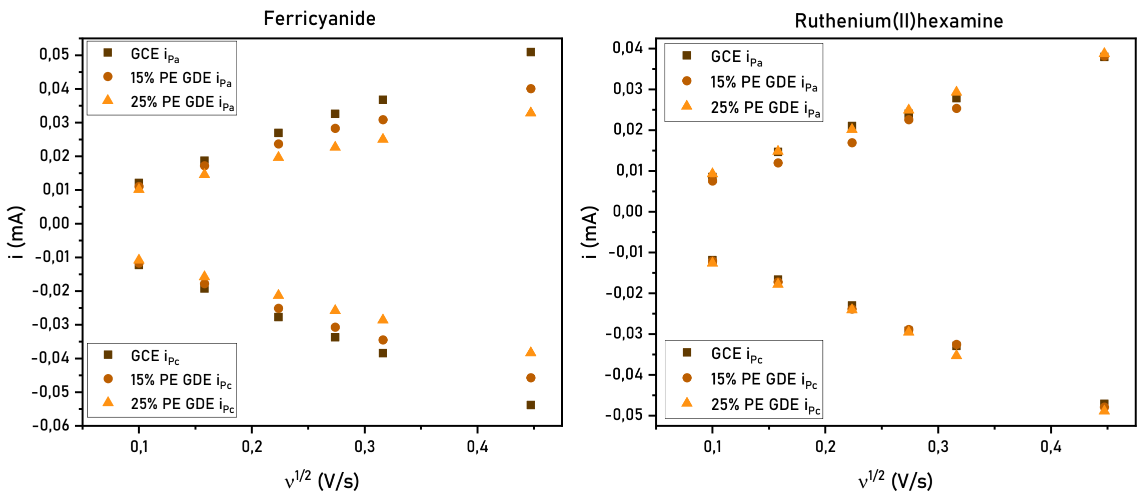

Figure S10. Plots of the linear dependence of the peak currents to the square root of the scan rate, comparing 15% PE, 25% PE GDEs and GCE.

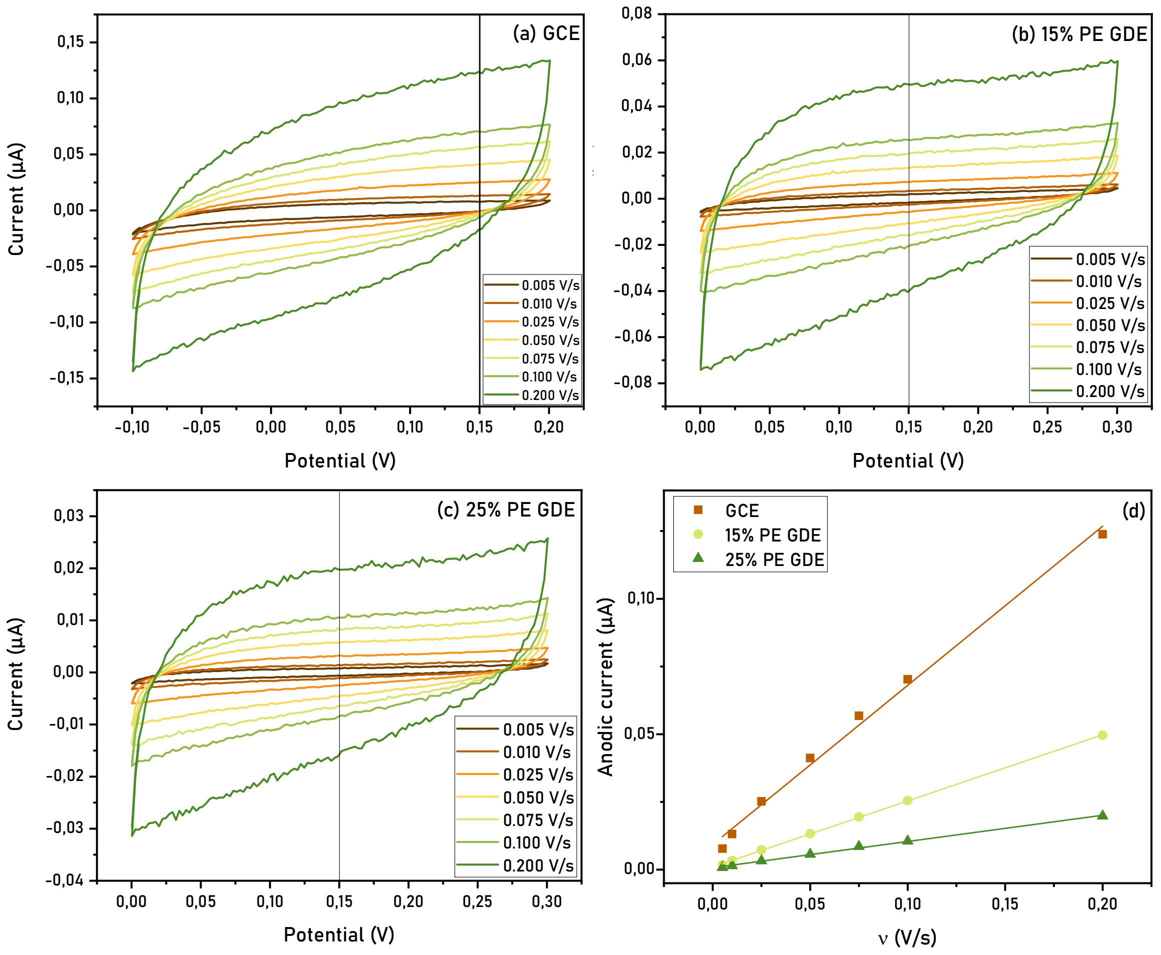

Figure S11. Double-layer capacitance measurement for (a) GCE, (b) 15% PE GDE, (c) 25% PE GDE; (d) linear dependence of the anodic current at 0.15 V to the scan rate for all electrodes in MES buffer.

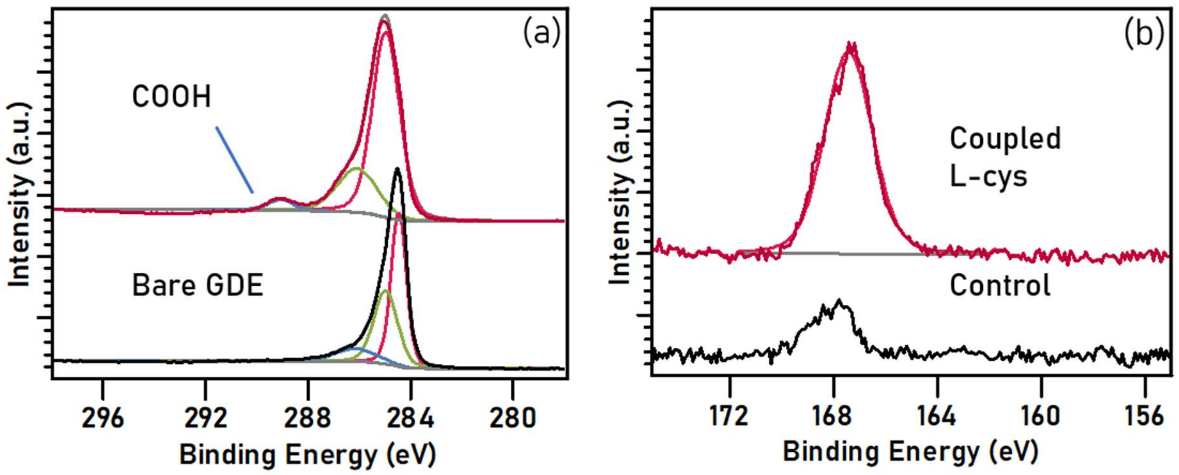

Figure S12. (a) C 1s spectra before and after electrode functionalization with 4-phenylacetic moieties; (b) S 2p spectra after covalent coupling of L-cysteine, compared to dropcasted solution.

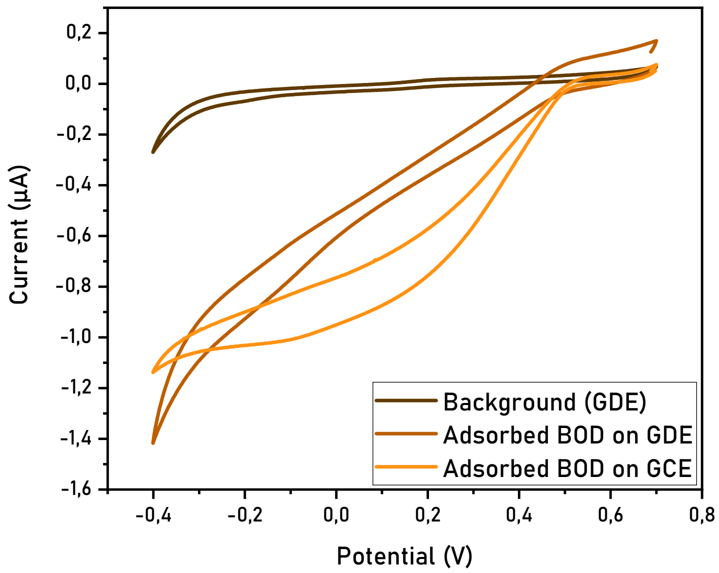

Figure S13. Comparison of dropcasting of BOD on GDE and GCE.

$$Fc \leftrightarrow Fc^{+}$$

$$2Fc^{+} + GODH_2 \rightarrow 2\,Fc + GOD + 2H^{+}$$

$$GOD + glucose \rightarrow GODH_2 + gluconolactone$$

Scheme S1. Mediated electron transfer process in presence of glucose oxidase.

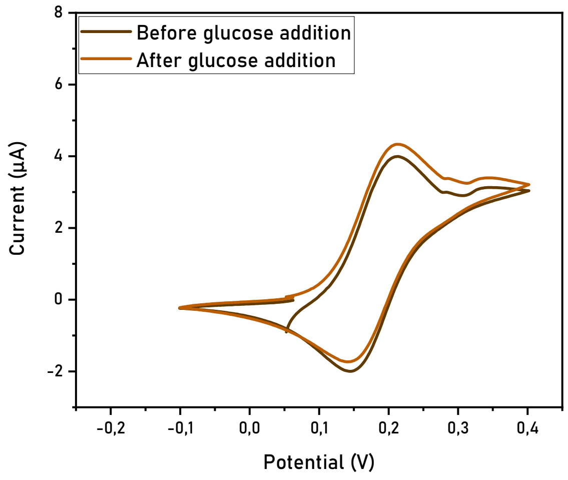

Figure S14. GOD adsorbed on carboxyl functionalized GDE upon glucose addition.

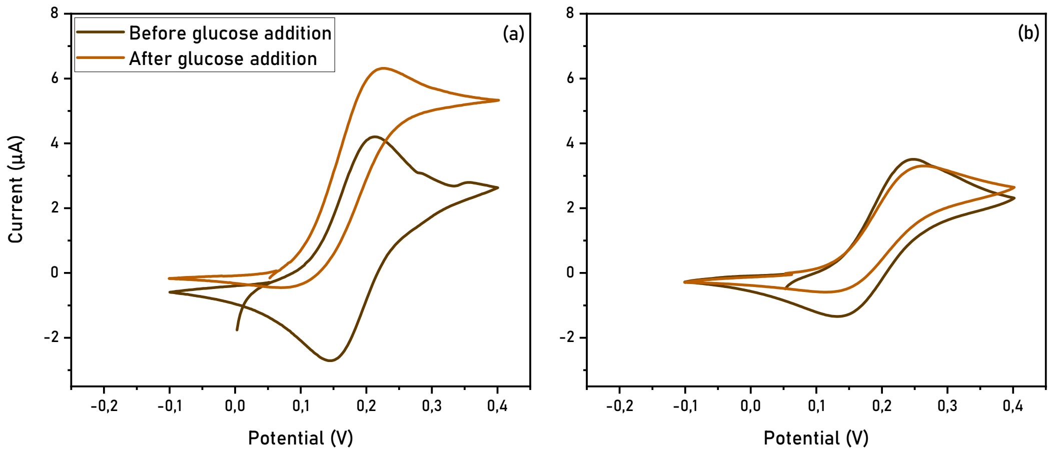

Figure S15. GOD (a) covalently coupled and (b) adsorbed on carboxyl functionalized GCE upon glucose addition.

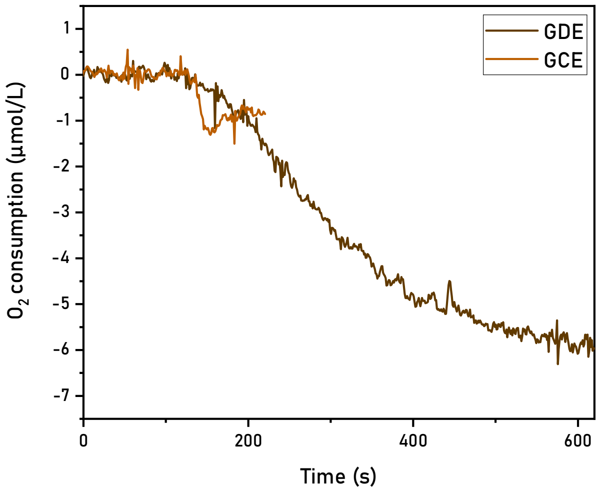

Fig S16. O_2 consumption measurement with Clark-type electrode upon addition of glucose, comparing covalent coupling of GOD on GDE and GCE.
